# “An Unusual and Fast Disappearing Opportunity”: Infectious Disease, Indigenous Populations, and New Biomedical Knowledge in Amazonia, 1960–1970

**DOI:** 10.1162/POSC_a_00255

**Published:** 2017-09-29

**Authors:** Rosanna Dent, Ricardo Ventura Santos

**Affiliations:** McGill University; Fundação Oswaldo Cruz and Museu Nacional/UFRJ

## Abstract

In the twentieth century, biomedical researchers believed the study of Indigenous Amazonians could inform global histories of human biological diversity. This paper examines the similarities and differences of two approaches to this mid-century biomedical research, comparing the work of virologist and epidemiologist Francis Black with human geneticists James V. Neel and Francisco Salzano. While both groups were interested in Indigenous populations as representatives of the past, their perspectives on epidemics diverged. For Black, outbreaks of infectious diseases were central to his methodological and theoretical interests; for Neel and Salzano, epidemics could potentially compromise the epistemological value of their data.

## 1 Introduction

In 1966, a team made up of Brazilian and foreign scientists spent a week carefully recording the body temperature and other clinical signs and symptoms of 110 Tiriyó Indigenous people in their communities along the Brazil-Suriname border (Black et al. 1969). Led by the Yale University virologist and immunologist Francis Black, the researchers faced an “epidemic” with a special profile, distinct from those most common in Indigenous populations, which usually resulted in widespread illness, the collapse of subsistence activities, hunger, and as a rule, elevated mortality (Ribeiro 1956; Coimbra 1987; Crosby 2003; Jones 2003, 2004).

Rather, what was happening with the Tiriyó was a planned event, controlled and carefully monitored. It was the result of the application of a new biomedical technology, the Schwarz live attenuated measles vaccine. Around the globe, the vaccine was being tested in human populations as part of diverse initiatives, including one by the World Health Organization (WHO 1963). But the field research led by Black was not only designed to test the vaccine. As Black would emphasize a few years later, the “vaccine makes a convenient, ethical model for the study of measles because it elicits almost all symptomatic aspects of the natural disease, but in less intense form” (Black et al. 1982, p. 42).

By monitoring the clinical conditions of the Tiriyó, the scientists hoped to confirm that the vaccine was safe for Native populations of the New World. Measles vaccine development initiatives of the 1960s were investigating the production of both inactivated virus vaccine and use of attenuated strains. In this context, a major debate emerged regarding whether Indigenous populations would be immunologically “competent.” Would they produce antibodies once exposed to the attenuated live virus similarly to Old World populations with long histories of exposure? Or were their immune systems insufficient to stop the attenuated strain, leading them to develop the disease that was supposed to be prevented? Black’s research team concluded that the Tiriyó had reactions to the vaccine that implied “no serious impediment to their immunization” (Black et al. 1969, p. 174).

The Tiriyó research was Black’s first foray into Amazonia. Over the next four decades, his fieldwork there would grow into a central focus of his scientific career (Black 1994, 1997; Santos 2015). Black’s research would begin with the data he collected in the context of vaccine field trials, as part of a global program that aimed to reduce measles epidemics. Using the serological profiles of the Tiriyó and others, Black would then turn his attention to the relationship between demography and epidemiological realities, theorizing the relationship between key parameters, such as population size, and the persistence or absence of certain kinds of pathogens (Black 1975; Black et al. 1974). His shift to these broader questions of what kinds of infectious diseases—viral, parasitic, bacterial—could persist in human groups with specific population densities and structures was explicitly linked to efforts to understand human evolutionary history and the rise of agriculture and “civilization” as related to epidemiological realities in the present. Considering diseases like measles that were virulent, fast-spreading, and short-lived, Black theorized that certain infections could only have been sustained in human populations following the advent of agriculture and the resulting agglomeration of populations of large size and density. It was in this context that, a few years after beginning his studies in the Amazon, Black would write, “contemporary primitive societies perpetuate conditions which existed in ancient peoples” (1975, p. 515).

One of the principal biomedical scientists to work in Amazonia in the second half of the twentieth century, the Yale researcher was part of a generation that turned to study the biology of Indigenous people. They conducted research in the most diverse parts of the world, with a view to developing genetic and epidemiological models applicable to the human species. The mid-twentieth century scientific narratives that emerged from this kind of work positioned Indigenous populations as “portals to the past” (Radin 2013, p. 487). These approaches gained popularity from the 1960s on, especially in the field of human population genetics. Researchers conceived of Indigenous populations as privileged subjects, essential to understand broader processes of human evolution on time scales in the tens of thousands of years (Santos 2002; Lindee and Santos 2012; Radin 2013, 2017; Kowal et al. 2013; Santos et al. 2014; also see Haraway 1989).

However, scientists’ epistemological privileging of Native groups not only sought to inform understandings of the past; they were narratives fundamentally concerned with the future (Radin 2013; Kowal et al. 2013). In the context of the escalating Cold War, Indigenous populations were assumed to be unexposed to the growing levels of radiation observed in the West. As such, the study of Indigenous population genetics was seen as potential sources to inform understandings of “natural” levels of mutation and genetic change resulting from technological change (Lindee 2001, 2004; Bangham and de Chadarevian 2014; Santos et al. 2014). According to historian of science Joanna Radin, in the context of research programs such as the International Biological Program (IBP), an important portion of international biological diversity research in the 1960s to 1970s was predicated on the idea that Indigenous “…bodies preserved traces of the deep past, which could have potential value for the survival of humans into the deep future” (Radin 2012, pp. 89–90). Indigenous populations were conceived of as “living closer to, and in balance with, nature” (Radin 2012, p. 70), and as such, they could serve as models both for the past and the future.

Recent scholarship has shown how biomedical researchers in the 1960s and 1970s justified the privileged epistemic position of Indigenous populations as subjects for study by emphasizing specific geographical, cultural, and ecological characteristics and providing salient interpretations of human temporality. In this paper we build on this work by inquiring into the potential and problematics that infectious disease posed for scientists’ approaches to Indigenous populations of the Amazon. Disease, whether endemic or introduced, epidemic or consistently present at a low-grade level, became an important variable in establishing how useful certain communities could be for scientific investigation. Infectious disease had particular relevance, as scientists understood susceptibility and resistance to be essential determinants of genetic “fitness” and the probability of individuals and communities to propagate their genes. Indigenous communities’ status as “Populations of Cognition” hinged on their health profiles and the presence, absence, or prevalence of particular diseases—a key factor that has yet to receive due attention (Suárez-Diaz et al. 2017). Indigenous groups functioned as populations of cognition by allowing scientists to conceptualize or model scientific problems; researchers used Indigenous populations as representational tools to understand large-scale processes at the level of the human species. They projected contemporary observations into the past, ascribing meaning based on various assumptions about the groups they studied in order to understand past, present, and future. Quantifying and interpreting the natural history of infectious disease had substantial implications for the validity of their scientific claims.

In this paper, we address the complex interaction between the presence of infectious disease and scientists’ claims about temporality and the representative nature of Indigenous subjects. In the first of two parts, we present an historical contextualization of human biological diversity studies at the beginning of the 1960s. Disease profiles were of great interest to researchers who traveled to Amazonia during this period; predominantly, they sought the most isolated and pristine (“primitive”) populations possible as a key to the “deep past” and the “deep future.” Focusing on the collaborative work of geneticists James V. Neel of the University of Michigan and Francisco M. Salzano of the *Universidade Federal do Rio Grande do Sul*, Brazil, we show how this approach required groups perceived as minimally “contaminated” by surrounding national society. On the one hand, researchers were interested in the interface of genetics and disease, especially those diseases understood as predating European colonialism. On the other hand, introduced disease was seen as potentially “polluting” analytical models. Their studies required “purity,” in the sense of populations that could be portrayed as being as close as possible to what Neel would call “the conditions of human evolution,” unchanged by colonialist expansion and uncontaminated by Old World disease. In the second section, we analyze Black’s research in Amazonia. In contrast to other researchers, between 1966 and the mid-1970s, Black and his collaborators focused primarily on introduced diseases.^1^ While notions of “the primitive” and the conceptualization of Indigenous communities as “portals to the past” were important guiding axes for Black’s research, his approach took a distinct angle from many of his colleagues. We argue that Black’s models of human evolutionary history, epidemiology, and demography were also imbricated with the “deep present,” in the sense that the occurrence of epidemics related to the expansion of settler colonialism into the Amazon played a key role in his theoretical constructions.

## 2 The Purity of the Deep Past

Beginning in the 1960s, there was a notable expansion of biomedical research on Indigenous populations from the Amazon to Polynesia, the Arctic to Africa (Santos 2002; Reardon 2005; Anderson 2008; Radin 2013, 2017; Kowal et al. 2013). At the core of this interest was the perspective that primeval and pristine, Indigenous groups’ biology could illuminate human evolutionary history. In the context of growing concerns about the annihilation of the human species due to emergent technologies – particularly atomic ones – and environmental change, Indigenous peoples’ biology offered “lessons” according to James Neel in his well-known *Science* article “Lessons from a ‘Primitive’ People” (Neel 1970; see also Santos et al. 2014). Subtitling his article, “Do recent data concerning South American Indians have relevance to problems of highly civilized communities?” the geneticist answered his own question with a firm “yes” based on his research in Amazonia.

Within the international scientific community, Neel was particularly successful at promoting what he saw as the research potential of Indigenous communities. Working closely with the World Health Organization, his 1962 field collaboration with Brazilian geneticist Francisco M. Salzano and three other scientists became a model for later larger-scale research initiatives (de Chadarevian 2015). The two geneticists began developing the theoretical foundation for their joint research in the late 1950s while Salzano completed Rockefeller Foundation funded post-doctoral training in Neel’s laboratory in Ann Arbor. Their first foray into the field took them to the Xavante of Central Brazil (Neel et al. 1964; Neel 1994; Salzano 2000; Dent 2017). This initial research, conceptualized as a pilot project, grew into a sustained collaboration that continued until the middle of the 1970s and included various other populations, particularly the Yanomami along the Brazil-Venezuela border (Neel 1994; Salzano and Callegari-Jacques 1988).

Neel and Salzano’s collaboration had implications both at the level of national science in Brazil as well as in high profile, international research initiatives. Salzano was one of a number of young Brazilian geneticists to complete fellowships in the United States in the late 1950s and early 1960s, just as human genetics emerged as a field in Brazil (Souza and Santos 2014). Beyond genetic analyses of the general formation of the Brazilian population, studies on biological diversity of Indigenous populations, particularly those located in Amazonia, became one of the pillars of the newly institutionalized field in Brazil (Salzano 1991, 2000; Souza and Santos 2014; Dent and Santos 2016). The focus on “primitive” people likewise became a prominent, widely disseminated approach to the study of human diversity. The study of Indigenous groups was institutionalized as a methodology for the study of human evolutionary history in the worldwide research initiative known as the “Human Adaptability” arm of the International Biological Program (HA-IBP) (Santos 2002; Lindee and Santos 2012; Little 2012; Radin 2013).

The Amazonian research of Neel, Salzano, and their collaborators resulted in the publication of dozens of papers, which, with varying levels of impact, addressed questions linked to the occurrence of disease (for a general vision, see Neel 1994; Salzano and Callegari-Jacques 1988). Their general approach to the analysis of infectious disease was already apparent in their earliest work at the beginning of the 1960s.

As previous scholarship has shown, Neel and Salzano vigorously promoted the notion that the “American Indian” posed particular “genetic and para-genetic questions” (Neel and Salzano 1964, p. 85; see also Santos 2002; Radin 2013). “Pre-eminent” among “surviving primitive groups,” they wrote, American Indians “present an unusual and fast disappearing opportunity to study the selective forces which shaped modern man” (Neel and Salzano 1964, p. 91). Focusing on the scientific potential of groups that were thought to descend from a single founding population that initially settled the American continent “some 30,000 years ago” (Neel and Salzano 1964, p. 85), the scientists thought they would be able to illuminate the forces of selection that had been at play for “the majority of human evolution” which “occurred under conditions far more comparable to those to be observed in the surviving primitive groups of the world than in today’s major culture-complexes” (Neel and Salzano 1964, p. 91).

From the perspective of the geneticists, the “genetic problems posed by the descendants of these migrants” included three principal sets of questions: (1) “what is the degree of genetic divergence which has arisen between the various tribal subdivisions of the descendants of the one or more founding stocks?”; (2) “For such of these groups as still persist in an essentially pre-Columbian state, to what extent can we identify the significant biological parameters, parameters which we may presume to have obtained over the majority of human evolution?”; and (3) “What new disease patterns will emerge as these primitive groups make the transition from a near-Stone Age to an Atomic Age existence?” (Neel and Salzano 1964, p. 85).

In their early work together, the geneticists focused on the second question: their primary objective was to illuminate the mechanisms—both biological and social—underlying the production of genetic variability in human populations (Neel and Salzano 1967, p. 555). In order to do this, they proposed the collection of a wide range of biological, anthropological, and demographic data. This included morphological and descriptive genetic data; “data on those aspects of the cultural pattern with biological implications”; “data on population structure, the term including birth and death rates…age distribution, inbreeding, migration…”; and “data on psychological pressures, with particular reference to the relation they bear to survival and reproduction” (Neel and Salzano 1964, p. 91). The study of disease was a component, not for its own sake, but for its potential to inform the relationship between evolutionary pressure and genetic variability. This included investigations into infectious and parasitic diseases and nutrition status, which the geneticists saw as selective pressures, but excluded attention to non-infectious diseases such as cancer or diabetes, unless readily observable in physical exams. Neel and Salzano described as much, advocating the collection of, “Data on biological pressures, the term including exposure to agents of diseases and the manner of acquisition of immunity as well as an evaluation of elements in the diet which when deficient or in excess are disease producing” (Neel and Salzano 1964, p. 91).

At the same time that the researchers emphasized disease as a relevant element, under certain conditions disease histories and prevalence created difficulties for the geneticists’ analytic models. Reporting on their 1962 pilot study of one Xavante village, Neel, Salzano, and colleagues wrote that “attempts to depict the disease profiles of such groups as the Xavante” were necessary both for their “contribution towards defining the disease pressures under which primitive man evolved,” and for diverse goals in human population genetics, including characterization of the role of genetic polymorphisms in determining disease resistance (Neel et al. 1964, p. 117). Due to the “biological pressures” exerted by disease, the researchers were particularly interested in pre-contact infectious diseases—those that had been endemic prior to the arrival of Europeans—and what these diseases could elucidate about the (genetic) “manner of acquisition of immunity” (Neel and Salzano 1964, p. 91).

However, non-endemic epidemic diseases could potentially destabilize the factors that made Indigenous populations of such interest by undermining the possibility of their population structures “persist[ing] essentially in a pre-Columbian state” (Neel and Salzano 1964, p. 85). The geneticists freely admitted these limitations in their earliest publications. Discussing their 1962 pilot study, they wrote that the observed high rates of antibodies to diseases like measles and pertussis “could be evidence for serious epidemics among the inhabitants of this village in the recent past. If so, this casts serious doubt on the validity of our demographic data as a representation of the primitive state” (Neel et al. 1964, p. 128). In other words, if the observed groups had a history of recent epidemics of Old World diseases, this would contaminate the data, undermining the epistemological system that posited Indigenous groups in the present as a window into the past. Yet they persisted in their optimism that the Xavante and other Indigenous groups could and would inform understandings of human evolution, citing the need for more and more extensive studies.

From the perspective of the geneticists the occurrence of disease was a constitutive and unequivocal part of human biological experience, a fundamental context for evolutionary processes over millions of years (Neel 1994; Neel and Salzano 1964). In selecting “primitive groups” to study, the key issue was not the absence of disease; the geneticists recognized this as an impossibility. Rather they looked for subject populations “persisting in a relatively ‘unspoiled’ form whose economy was based on hunting, gathering, and incipient agriculture” (Neel and Salzano 1964, p. 91). In large part, to be “unspoiled” meant to have avoided interaction with encroaching settler society and the devastating concomitant changes in economic, demographic, and epidemiological status.

In their 1964 “Prospectus for Genetic Studies of the American Indian,” Neel and Salzano included a list of “some surviving primitive groups in the Americas” that could be studied according to their research agenda. They included those populations they believed to be most “unspoiled.” Less than believing that Indigenous populations were in fact isolated and pristine, scientists like Neel and Salzano recognized that, with more or less intensity, the variables that they were recording could have already been influenced by the historical, demographic, and socio-political context these groups had been plunged into through interaction with non-Indigenous people.^2^ However, Salzano and Neel’s methodology of Indigenous-population-as-time-portal drew its authority in part from the absence of introduced disease, which allowed them to draw links to a pre-Columbian past. Documenting and promoting the “excellent physical condition” of groups like the Xavante, Yanomami, and Makiritare (Neel 1970, p. 818) allowed the scientists to make truth claims about the representative nature of the groups they studied and the insight their methodology could offer for knowledge of the deep past.^3^

## 3 The Contamination of the Deep Present

Neel and Salzano, whose research program situated disease as a coadjuvant agent for their primary interest in human genetic change, spent years considering collaborative fieldwork and months planning the details of their first joint expedition to Amazonia in 1962. In contrast, Francis Black’s first trip to Brazil was abrupt and unexpected. It was prompted by a telegram he received in 1966 from Jack Woodall, a researcher at the Rockefeller branch laboratory in Belém, in the state of Pará (Black 1997, p. 37). From the beginning of his work in the Amazon, Black’s interests were distinctly ordered from those of the geneticists: infectious diseases were the variables to be explained, with genetics, among other factors, occupying a potentially explanatory role.

Woodall had participated in an expedition to the Tiriyó, in the north of Amazonia, and the analysis of the collected blood samples indicated that, in general, the population had not been exposed to measles (Black 1997, p. 37). Having spent time at Yale, Woodall was up to date on Black’s research on the immunology of measles exposure and responses to measles vaccination in other areas of the world. In just a few weeks after receiving the invitation, Black prepared for what would be his first trip to Amazonia with the goal of testing “measles vaccine reactions in a virgin [soil] population” (Black et al. 1969).^4^

In the 1960s, Black played an important role in the studies that led to the first measles vaccines (Black 1997; Santos 2015). Already at Yale in the second half of the 1950s, he drew on his training in chemistry and virology to develop epidemiologic studies of immunological markers in order to estimate the scope and periodicity of epidemics. From this early portion of his career, which primarily focused on field research in the United States (Black 1959, 1997), Black increasingly turned his attention to the rest of the world. In 1963 he participated in a WHO-hosted meeting of measles vaccine specialists (WHO 1963), and in the context of the development and tests of the first measles vaccines he shifted more definitively to international research (Black and Rosen 1962; Black and Gudnadóttir 1966; Niederman et al. 1967).

In tests of the vaccine, it was common to try to find a population that, at least in recent decades, had not suffered measles epidemics as shown through the absence of antibody titers against the virus. In the early 1960s, scientists were testing diverse strains of vaccines, some based on inactivated virus (i.e., dead virus), and others based on live attenuated virus (i.e., virus that had been repeatedly cultured in specific media in the laboratory in order to reduce its virulence). After a long series of laboratory studies, developers needed to test the safety of a vaccine in human subjects to ensure it provoked the lowest possible clinical reaction while simultaneously conferring a high level of immunity.

Beyond simply focusing on baseline clinical and immunological responses of vaccinated individuals, Black was interested in the relationship of these baselines to the demographic, epidemiological, and genetic profiles of human populations (Black and Gudnadóttir 1966). As a disease that conferred life-long immunity, Black reasoned that measles could only have developed recently in human history, possibly in the last ten thousand years, when large populations on the order of thousands of people began to form. This demographic profile accompanied the emergence of agriculture in a number of regions around the world, in particular the “great valley civilizations.” Why was the evolution of the virus dependent on the formation of such large groups of people? The argument was that these conditions would allow for the continuous circulation of the virus, with new infections occurring due to the consistently renewing pool of unexposed individuals, especially children. Thus, Black reasoned, beyond simply being a recent addition to human history, there would be populations in the world that had never had contact with the infection. This would be the case, for example, for some communities of Indigenous peoples of the Americas, whose ancestors would have arrived on the American continent thousands of years before the infection established itself as a disease of human populations. Those groups with extremely limited contact with settler society, Black imagined, could have avoided contamination.

In the vaccine field trials, which were focused on “vaccine safety, efficacy and applicability” (WHO 1963, p. 5), Black saw an opportunity to inquire into his theoretical formulations on the natural history of measles. His ideas were strictly associated with specific applications, such as the debates regarding the appropriateness of applying a vaccine developed with live virus in a human population that had never before been exposed to this pathogen. In this context of the strict interface of the theoretical and the practical, Black began to conduct studies that conceptualized the “live vaccine as a model for measles” (Black et al. 1971).

According to this line of thinking, one of the most important aspects was to locate populations that, due to their history of isolation, had not been exposed to epidemics of measles, or if exposed, then only in the distant past such that individuals no longer showed immunity in the form of antibodies to the virus. One of the first studies by Black on what he called “populations at special risk” was based on samples from Tahiti, in Polynesia (Black and Rosen 1962). The scientist also conducted research in Iceland, testing various different kinds of measles vaccines in field trials. It was not a coincidence that these studies were conducted on islands, in so-called “insular populations” (Black 1966), since they were perceived to guarantee these populations’ high level of isolation.

Likewise, the study of Indigenous populations in the Americas was of particular interest to Black, since scientists generally accepted the hypothesis that measles had been introduced in the region with the arrival of Europeans at the end of the fifteenth century (Black et al. 1982). Over centuries of European expansionism, the majority of American Indigenous populations had already been exposed. Nonetheless, the most remote regions only recently subject to colonization as of the 1960s, still had relatively isolated Indigenous groups. It was for this reason that Black imagined he could find “virgin soil populations” in the Amazon (Black 1975; Black et al. 1982). From the perspective of the scientist, certain Indigenous communities could be seen as “insular populations,” isolated not by oceans, but by vast tropical forests:

Isolation by wide stretches of water has never been typical of the majority of mankind, and failure of certain diseases to persist in the islands had not been seen as relevant to the condition of the human race in general. In fact, the isolation of primitive mainland groups from one another may be as profound as the isolation of island populations… (Black 1975, p. 516)

Even if the initial motivation was to investigate reactions to the measles vaccine in Indigenous Amazonian populations, Black expanded the spectrum of his research agenda almost immediately (Black et al. 1982). As development projects and the growing occupation of Amazonia by non-Indigenous Brazilians resulted in Indigenous groups’ violent exposure to the frontier (Coimbra 1987; Hemming 1987; Carneiro da Cunha 1992; Ramos 1998), it became increasingly difficult to locate populations unexposed to epidemic infectious disease, including measles.^5^ It was in this context that, increasingly, the scientist’s attention turned away from specific infections and towards modeling the relationship between demography and the persistence of infectious disease (Black 1975; Black et al. 1974).

Black had already laid the empirical foundation for this broadening of his research focus in the original study with the Tiriyó (Black et al. 1970). The approach drew on a theoretical orientation that Black had articulated in the mid-1960s regarding the role of demographic factors such as population size and density in determining whether infectious disease could be endemic (Black 1966). Specifically, after the first study in the Amazon, Black and his team performed immunological tests not only for resistance to the measles virus, but also, “the prevalence and distribution of antibodies against 38 different viruses,” including influenza, mumps, rubella, and poliomyelitis (Black et al. 1970). Even though in principle they were studying a narrowly circumscribed case, the authors offered the justification that their broad approach would inform questions relevant to human evolution.

Based on the approximately 180 Tiriyó samples collected and analyzed in 1966, Black and his collaborators did not arrive at theoretical generalizations regarding human evolutionary history. Rather, they began a longer process, affirming that:

The present serum collection offered an opportunity to identify some of the viruses which could perpetuate themselves under these conditions and, hence, might be implicated as possible ecological elements in the development of the human species. (Black et al. 1970, p. 430)

By the mid-1970s, the accumulation of a decade of data from diverse field research, primarily in the state of Pará, allowed Black and collaborators to publish a more comprehensive, comparative study titled “Evidence for Persistence of Infectious Agents in Isolated Human Populations” (Black et al. 1974; see also Black et al. 1971). In addition to analyzing a vast array of viruses, the scientists broadened the gamut of infectious agents, including tuberculosis, malaria, tetanus, and filariasis. According to the authors,

The purpose of this study is to determine which of our modern disease agents are able to persist in small, isolated communities… They offer a further advantage in the reconstruction of man’s heritage of disease, in that they are still hunters and gatherers, as were all men through most of their evolutionary history. (Black et al. 1974)

In essence, the scientists developed the argument that under the demographic conditions of small, isolated populations, parasitic infections would persist as would long-term, contagious viral infections. Other kinds of infection, especially those that spread rapidly and provoked strong immunological reactions that conferred lifelong immunity would not persist due to the small pool of potential carriers. Seropositivity (antibody titers) by age constituted a principal point of analysis, indicating whether or not infections had occurred recently based on whether they were concentrated in specific age groups. If only one part of a population showed seropositivity, (the eldest, for example), it indicated the absence of continuous transmission, potentially the result of a period of contagion years or decades earlier.

It was based on this kind of evidence that the scientists argued that the herpes and hepatitis B viruses were endemic in the Indigenous populations analyzed, “maintain[ing] a very stable relation with their host populations” (Black et al. 1974, p. 230). This occurred because these “disease agents spread very effectively in these closely knit communities” (Black et al. 1974, p. 248). On the other hand, there were infectious agents such as those that caused measles, rubella, and influenza, which were characterized by “unstable relations…appearing only when introduced from the outside” (Black et al. 1974, p. 230). These diseases had to be introduced from areas of much greater human density, where the population was large enough to sustain continuous infection.

The analytic scope of Black et al.’s 1974 paper went beyond characterizing the serological profile of a group of Amazonian communities. Detailing the findings according to “epidemiologic patterns”, the authors generalized about the relationship between origins and dispersion patterns of infectious diseases on a broader scale. This is clear in their comments on smallpox: “the absence of any evidence of smallpox… may mean that the virus evolved since the dispersal of these people from the Old World centers…” (Black et al. 1974, p. 246). They also suggested that malaria and tuberculosis were not endemic to the New World, but rather were introduced with the arrival of Europeans.

**Figure 1:**
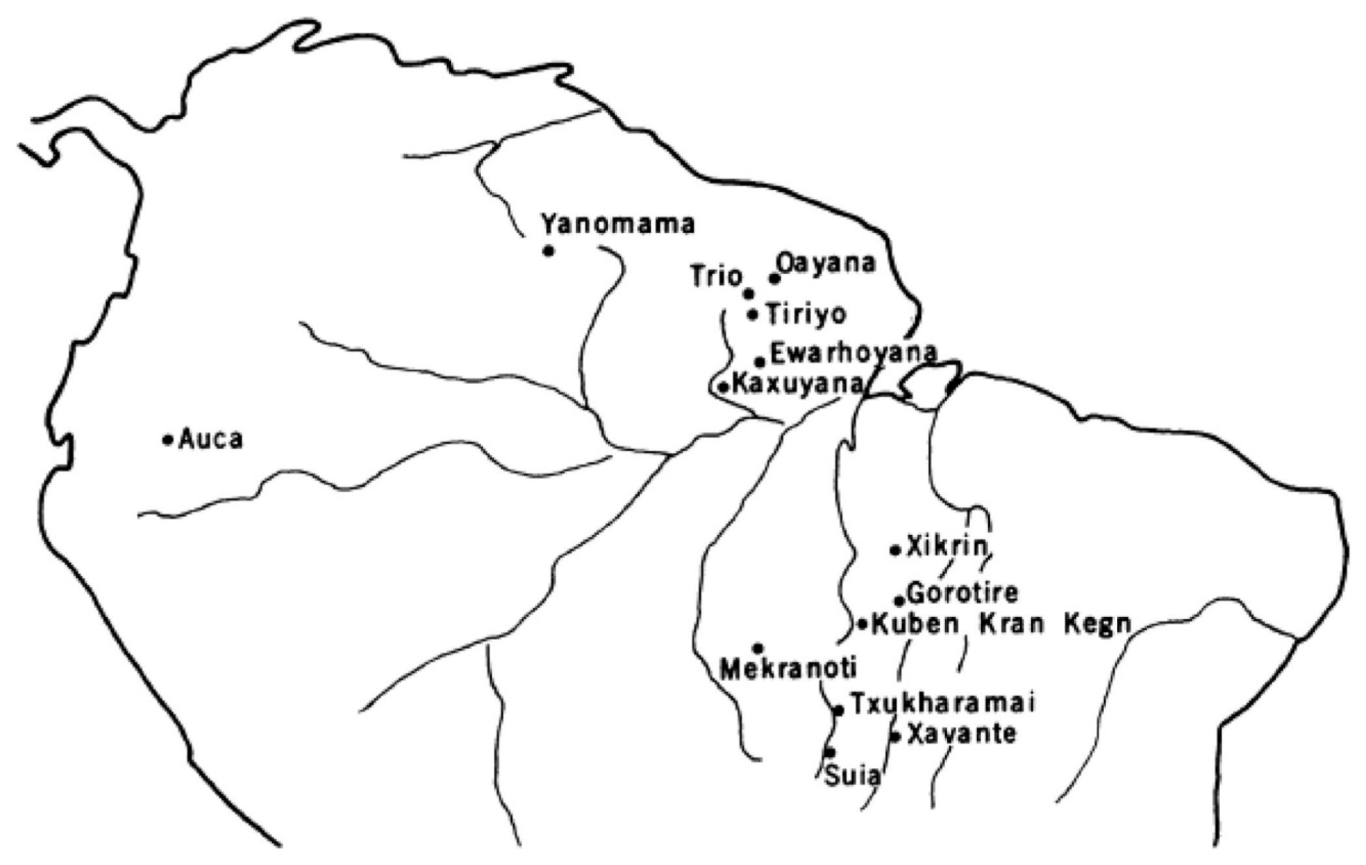
“Sketch map of northern South America indicating the locations of the tribes referred to in the article” (Source: Black 1975, p. 516).

In 1975, approximately a decade after his first fieldwork with the Tiriyó, Black published an article in *Science* that would come to be one of his bestknown works. Black offered two general conjectures. First, he related the importance of infectious disease to the evolutionary history of the human species, writing, “Infectious diseases have exerted some of the strongest of the pressures that shaped the development of modern man” (Black 1975, p. 515). Black emphasized the notion that Indigenous populations could serve as “models” in the understanding of processes related to epidemiology and human evolutionary history. In “Infectious Diseases in Primitive Societies” Black presented the data from his Amazonian studies (Figure 1), complemented by those of other authors, including Neel and Salzano, who had published seroepidemiological studies based on Amazonian samples.^6^

The 1975 *Science* article makes clear the extent to which potentially epidemic disease was central to Black’s theoretical approach. This contrasts with the predominant approach of population genetics, as we saw in the last section, which as a rule understood epidemics to negatively affect their model building by causing demographic transformations that impacted genetic parameters. According to Black, serological analyses of Amerindian samples could potentially inform the reconstruction of human epidemiological history:

Unless ancient conditions were fundamentally different from those of surviving primitive cultures, measles, influenza, smallpox, and poliomyelitis could not have been present during the period of human emergence nor through most of the man’s history. (Black 1975, p. 518)

For Black, Indigenous populations were a window into the natural history of disease in human populations. His notion of the “deep past” was different from that of the geneticists in important ways. For the geneticists, the lifestyle of hunter-gatherer was of utmost importance, as it was the predominant life-mode for the vast majority of the human evolutionary trajectory and therefore maintained a continuity of selective pressures on the gene pool (see Neel and Salzano 1964; Neel et al. 1964). From Black’s perspective, a more recent “deep past” was also central, and understood Amazonian Indigenous groups to represent the “second phase of human development, with incipient agriculture and relatively settled villages …” (Black 1975, p. 516). For the virologist, it would have been under these conditions during the previous thousands of years that many human infectious diseases had established themselves. Furthermore, the recent history of contagion through contact with Old World diseases and members of the dense, populous communities that sustained them added important evidence for his generalizations about the nature of epidemic diseases. As he concluded in his 1975 text, diverse infections that presented as epidemics in the contemporary world “could not perpetuate themselves before the advent of advanced cultures and did not exert selective pressures on the human genetic constitution until relatively recently” (Black 1975, p. 518). In Black’s approach, Indigenous populations were useful because their small size and relative isolation meant that disease distribution and persistence in the present could inform understandings of the past.

## 4 Concluding Remarks

In this paper, our comparative analysis highlights the simultaneous convergences and heterogeneities of scientific thinking about temporality and disease in mid-twentieth century research with Indigenous people. Both the geneticists and the virologist-epidemiologist were interested in the relationship between human genetics, evolutionary theory, and the epidemiology of infectious disease, but with different emphases. Indigenous populations, as Populations of Cognition (see Suárez-Díaz et al., this issue), served different ends. For Neel and Salzano, these populations allowed them to think in certain ways about how biological and socio-demographic factors influenced the production of patterns of human genetic variability, with disease understood as part of the pool of possible “selective pressures.” For Black, the study of Indigenous populations allowed him to inquire into the relationship between the epidemiology of infectious and parasitic disease in relation to demographics. Based on his observations in these specific populations he proposed general models regarding the interrelated nature of human evolutionary history and the persistence or lack thereof of various groups of pathogens.

Despite differences in underlying interests, both approaches identified Native people as “representational tools” or Populations of Cognition for understanding the past, as well as making sense of the present and preparing for the future. Researchers approached their work in the Amazon basin with optimism and hesitation, understanding their models to be dependent on documenting and understanding endemic and epidemic illness. In the context of vast ecological and socio-economic change that accompanied the quickening national societies’ invasion of Indigenous territories, specific communities could be regarded as more representative to inform broader theoretical models proposed by the scientists if their epidemiologic profiles matched particular assumptions about past and present, purity or contamination.

Community histories of health and illness were essential to both perspectives, defining in large part whether and how the scientists could generalize based on the populations they characterized. The geneticists focused on the perceived vitality of the groups they visited, drawing their authority to make claims about the deep past based on what they perceived as the lack of disease and socio-economic change. The absence of disease, in the geneticists’ approach, was a surrogate for measuring the stability and timeless nature of the populations they studied. It was evidence that their temporal assumptions about the deep past were valid; it allowed the groups they characterized to function as populations of cognition. In contrast, Black’s focus on unexposed populations soon gave way to the study of disease load and population profile as the virologist recognized the widespread exposure to introduced diseases that accompanied western expansionism and encroaching settler colonialism. Black used his study of Indigenous groups to understand the natural history of disease on evolutionary timescales, and to draw inferences about how infectious disease spread and persisted in a more recent past that included both Native and settler populations. In his vision, immunologic and genetic factors were related to epidemics of infectious disease, including measles, and resulted from permanent contact with the surrounding national society. Black investigated contemporary processes with the hope of developing more general models regarding the interface of epidemiology and infectious disease for the evolutionary history of the human species. At the same time that “purity” and “isolation” were constitutive elements of his analytical models, “interaction” and “contamination,” in large part resulting from the “deep present,” linked his thinking to the economic and demographic transformations underway in the Amazon. Although Black continued to understand his observations on the relationship between demography and disease in Indigenous communities to inform epidemiologic models for the past, his later approaches allowed for the contamination of contact.

## Footnotes

1 It is important to emphasize that our goal in this paper is not to cover the full spectrum of epidemiologic research on infectious disease in Amazonian Indigenous populations carried out in the 1960s–1970s. Black, Neel, and Salzano were certainly among the leading authors on the topic, but there were also a few other researchers working on specific diseases in the region (for overviews, see Salzano 1990; Coimbra and Santos 1992; Coimbra 1995).

2 It is worthwhile to highlight that the researchers were involved in diverse initiatives, some of which were sponsored by multilateral agencies like the Pan American Health Organization and the World Health Organization and sought to influence initiatives and policies of health care for Indigenous peoples in the Americas (WHO 1964; Neel 1968, 1974; Radin 2014). On the involvement of Black with indigenous right issues, see Santos 2015.

3 As Santos, Lindee, and Souza have argued, Neel’s drive to find the most remote, most “primitive” groups would take him to the Yanomami on the northern side of the Brazil-Venezuela border (2014). He would describe his research there saying “One of the unsought distinctions of my studies was to be among the Yanomama during a measles epidemic, very possibly their first ever” (Neel 1989, p. 817; see also Neel et al. 1970). Neel’s observations would lead him to reject the hypothesis that Indigenous Americans were “innately susceptible,” blaming the epidemiological profile of the “‘virgin-soil’ village” rather than “individual biology” (1989, p. 818), a perspective that contrasted to the virologist Black. Neel’s work with Yanomami communities in Venezuela and Brazil and his close collaboration with anthropologist Napolean Chagnon would later be subject to intense debate following the publication of the hotly contested journalistic exposé, *Darkness in El Dorado* (Borofsky et al. 2005).

4 Black and colleagues emphasized that the research would be the *first* performed with Indigenous populations of the American continent understood as “virgin soil populations.” Based on immunological assays that identified the presence or absence of antibodies, these populations were characterized as having no evidence of measles. The three prior studies performed on Indigenous people in Panama and with Inuit groups in Alaska and Canada had involved groups that “had been exposed to measles in the past” (Black et al. 1969, p. 169). For a critical perspective on the concept of “virgin soil populations” see Jones 2003, 2004.

5 Black et al. (1982) reviewed studies that they had conducted on reactions to measles vaccines in Amazonia as well as studies conducted by other researchers such as Roberto Baruzzi, João Paulo Vieira-Filho, and James Neel. They concluded that there were differences in immunological responses between Indigenous and Caucasian groups: “Considerable individual variation in reaction to vaccine virus was observed even within these [Amazonian] populations, but the average reaction in the Indian involved more fever and, for the same fever, lower antibody response than the same vaccine in Caucasians” (1982, p. 42). For Black, the reason for the observed differences were not clear, and could possibly be related to genetic factors or due to nutritional conditions that impaired immunological responses. In the following years, the scientist would increasingly argue in favor of genetic determinants of immunological response (Black 1992, 1994) in contrast to Neel and Salzano’s interpretations (see footnote 3, also Santos 2015).

6 It is worth noting that the principal non-Amazonian control was data emerging from Black’s studies of the population of New Haven.
